# Exploring the relationship between genetic and environmental influences on initiation and progression of substance use

**DOI:** 10.1111/j.1360-0443.2006.01694.x

**Published:** 2007-03-01

**Authors:** Tom Fowler, Kate Lifford, Katherine Shelton, Frances Rice, Anita Thapar, Michael C Neale, Andrew McBride, Marianne B M van den Bree

**Affiliations:** 1 Department of Psychological Medicine, Cardiff University Cardiff, UK; 2 Department of Psychiatry and Human Genetics, Virginia Commonwealth University Richmond, VA, USA; 3 Oxfordshire Community Mental Healthcare Trust Oxford, UK

**Keywords:** Adolescent, CCC model, initiation, progression, substance use

## Abstract

**Aims:**

To examine the genetic and environmental contributions to the initiation of use and progression to more serious use of alcohol, cigarettes and marijuana during adolescence, and to examine the relationship between initiation and progression of substance use.

**Design:**

The study used a twin-based design and a new theoretical model, the causal–common–contingent (CCC) model. This allows modelling of the relationship between initiation of use and progression to heavier use as a two-stage model and the examination of genetic and environmental influences on both stages, while taking into account their relationship.

**Participants:**

The participants consisted of 1214 twin pairs (69% response rate) aged 11–19 years sampled from the UK population-based Cardiff Study of All Wales and North-west of England Twins (CaStANET).

**Measurements:**

Data on adolescent initiation and progression to more serious use of alcohol, cigarettes and marijuana were obtained using self-report questionnaires.

**Findings:**

Initiation of alcohol and progression to heavier alcohol use had separate but related underlying aetiologies. For cigarette and marijuana use the relation between initiation and progression to heavier use was stronger, suggesting greater overlap in aetiologies. For all three substances, environmental influences that make twins more similar (common environment) tended to be greater for initiation, while genetic influences were stronger for heavier use.

**Conclusions:**

These findings have implications for policy decisions aimed at an adolescent and early adult age group. Specifically, these findings suggest that it may be more efficacious to focus alcohol interventions on risk factors for the development of heavier use rather than initiation of use. In contrast, interventions aimed at reducing the initiation of cigarettes and marijuana use may be more appropriate.

## INTRODUCTION

Experimentation with substances usually takes place during adolescence [1]. Adolescents are highly vulnerable to social influences [2], have lower tolerance levels and become dependent at lower doses than adults [3]. Adolescent-onset substance abuse is characterized by more rapid development of multiple drug dependencies and more severe psychopathology [4]. However, the majority of adolescents who experiment with substances do not become problem users. A better understanding is needed of the factors underlying initiation of substance use in adolescence versus heavy use and problem use. Specifically, if the liability to progress to heavier substance use is influenced by processes other than those that influence initiation, then primary prevention/intervention programmes can be only partly effective. It may be more successful, in terms of both cost and impact, to target those factors implicated in the progression to heavy/problem use. However, if the underlying liabilities to initiation and progression were strongly related, interventions could be tailored to both behaviours.

Tobacco use is associated with considerable economic, social and personal costs [5] and estimated to be involved in 4 million deaths world-wide each year [6]. Alcohol is the most prevalent form of substance use during adolescence [7] and marijuana is the most commonly used illicit drug by adolescents in both the United States [8] and the United Kingdom [9]. The negative consequences of substance use [10–12] have led to a significant research endeavour into the risk factors contributing to adolescent substance involvement, with the ultimate aim of developing the most effective prevention and intervention approaches.

Alcohol, cigarette and marijuana use during adolescence is a major cause for concern. The 2003 wave of the European School Survey Project on Alcohol and other Drugs (ESPAD) suggested that 91% of 15–16-year-olds in the United Kingdom have tried alcohol and 68% have been drunk within the last year [9]. This is higher than the European average for this age group (83% and 53%, respectively). Similarly, life-time prevalence of marijuana in this age group is also higher than the European average (38% versus 21%). Life-time use of cigarettes is somewhat lower (58% versus 66%); however, given the serious health outcomes associated with smoking, these prevalence rates are still of concern.

Genetic influences play a role in adolescent alcohol, cigarette and marijuana use [13,14]. Studies into the aetiology of substance use and abuse that do not take genetic influences into account may present an incomplete picture. The twin method is based on comparisons of monozygotic (MZ) twins, who share 100% of their genes, with dizygotic (DZ) twins, who share on average 50% of their genes. In the basic (ACE) model, variation can arise from three sources: (1) additive genetic effects (a^2^), (2) common environmental effects that are shared by twins and make them more similar (c^2^) and (3) unique environmental effects that are experienced by only one twin in a pair (e^2^) [15].

Twin studies can make an important contribution to understanding the relationship between the developmental stages of substance involvement [16]. In a seminal study by Heath and colleagues [17], the relationship between whether people have ever drunk alcohol and the quantity of alcohol consumed as well as the underlying genetic aetiology was explored with three separate conceptual models; the single liability dimension (SLD) model, the independent liability dimension (ILD) model and the combined model. The SLD model assumes a single underlying liability dimension, with abstinence at one end and heavy drinking at the other. The ILD model assumes two independent liability dimensions, the first determining abstinence/alcohol use and the second determining quantity of alcohol use. The combined model assumes that there are also two liability dimensions, positioned on the first determining whether a person is abstinent or influenced by the second liability dimension, and positioned on the second ranging from abstinence to heavy alcohol use. Research with adults has found support for this third ‘combined model’ for alcohol and tobacco use, with findings suggesting that common environment plays a larger role in whether an individual initiates drinking, whereas quantity of use is more genetically influenced [17–19]. However, these studies did not look directly at the relationship between initiation and progression.

Several recent studies have investigated the underlying aetiology of both initiation and progression to heavier/problem use of cigarettes, alcohol and marijuana in adolescence. Consistent with adult-based findings, most studies found a greater role for the common environment on initiation of substance use and greater genetic influence on heavier/problem use [13,14,19,20]. However, some studies reported greater genetic influences on initiation, dependent upon the substance investigated and gender (e.g. [13,14]). To date, twin studies of adolescent substance use have treated initiation and progression as independent constructs. Little is known about the underlying relationship between initiation and progression of substance use and the aetiology specific to progression in this age group.

A model [the causal–common–contingent (CCC) model] examining this relationship specifically and testing the degree of overlap between initiation and progression liabilities, as well as their genetic and environmental influences, was developed by Kendler and colleagues [21,22], and has been extended recently [23]. The model was designed to examine contingent data, i.e. where availability of information on a second variable (progression) is dependent on the response to an earlier variable (initiation). Thus initiation must, by definition, occur before individuals can progress to more frequent, heavy or problem use. Information on progression will therefore be available only for those who have initiated use. These types of data cannot be analysed with other more conventional models used frequently in twin studies, such as the Cholesky model or causal model [15].

Two twin studies have used this new method to examine the relationship between initiation and progression in cigarette use [24] and illicit drug use, including marijuana [25]. Both studies found a strong relationship between the initiation and progression stages of substance use. Of the underlying liability that was specific to the progression of substance use (after initiation had been taken into account), genetic factors were most important in cigarette use and unique environmental factors were most important in marijuana use. These papers used adult samples and, to our knowledge, no study has examined the relationship between initiation and quantity of use of cigarettes, alcohol or marijuana in adolescents. Although rates of alcohol and marijuana are among the highest in Europe [9], we are not aware of any adult or adolescent twin studies of either substance use in a UK sample.

Individuals vary in their response to the quantity of alcohol needed to become intoxicated [26] and metabolic rate of processing alcohol [27]. There are different behavioural consequences of higher blood alcohol levels and binge drinking (episodic heavy drinking), particularly in relation to increased aggression [28,29]. Furthermore, aggression increases with subjective perception of intoxication [28]. Indeed, there are specific risks involved in binge drinking and drunkenness that are separate from mean daily quantity of alcohol consumed [26,30]. It is therefore important, in addition to quantity of alcohol consumed, to look at a range of outcomes relating to problem use.

The aims of this study are to examine:

1. The prevalence of alcohol, cigarette and marijuana use in a UK population-based sample of adolescent twins.
2. The relationship between initiation and progression of substance use.
3. The underlying genetic and environmental aetiology for initiation and progression of use of these substances.

## METHODS

### Sample

The Cardiff Study of All Wales and North-west England Twins (CaStANET) is a population-based register of twin births between 1980 and 1991 in Wales and Greater Manchester, UK [31]. Information was used from the fourth wave (2004) of data collection on the substance use of the twins. A total of 1755 families with twins aged 11–19 years were contacted with mailed questionnaires. Non-responders were initially sent reminders, then remailed the questionnaire. Families received £15 ‘thank you’ payments in high street vouchers as a token of appreciation.

A total of 1214 families returned questionnaires (69% response rate). Participants were, on average, 16.11 years old (SD = 1.96). In the United Kingdom it is illegal to purchase cigarettes under the age of 16 and alcohol under the age of 18. Information was available for cigarette use on 1165 twins under 16 years of age and 861 twins aged 16 or over. For alcohol use, information was available on 1612 individuals under 18 years of age and 420 individuals aged 18 or over.

Zygosity was assigned in a previous wave of data collection [32], the sample consisting of 461 monozygotic (MZ) twin pairs and 714 dizygotic (DZ) twin pairs, with 39 twin pairs unassigned. The sample has been shown to be representative of the general UK population in terms of socio-economic status and ethnicity [31].

### Measures

#### Substance use

Levels of alcohol, cigarette and life-time marijuana use were assessed using questions based on the Add Health study [33].

Life-time use of alcohol and the quantity of alcohol drunk was assessed with the question: ‘Think of all the alcoholic drinks you had during the past 12 months. How many drinks did you have during a typical week? (A “drink” is a glass of wine, a can or half pint of beer or lager, a bottle (e.g. Bacardi breezer) or a single measure of spirits).’ Cigarette use was assessed with the question: ‘During the past month, on average, how many cigarettes did you smoke each day?’. Life-time marijuana use was assessed using the question: ‘During your life, how many times have you used marijuana?’. Possible responses to these questions ranged on a seven-point scale, from never having used the substance to using it more than 30 times.

#### Initiation

A binary initiation variable was created for each of the substances indicating whether an individual had or had not ever tried the substance.

#### Progression, alcohol

Quantity of use was indexed as light use (consuming between none and 10 alcoholic drinks during a typical week) and heavier use (consuming more than 10 alcoholic drinks during a typical week). This indicated whether the individual who had initiated had also progressed to heavier use. Progression to three other more serious alcohol use behaviours were also examined: binge drinking (having drunk at least five drinks in a row more than twice in the past year); getting drunk (having been drunk more than twice in the last year) and having been in situations later regretted due to alcohol (once in life-time). These were assessed with the following questions, respectively: (i) ‘Over the past 12 months, on how many days did you drink five or more drinks in a row?; (ii) ‘Over the past the past 12 months, on how many days have you got drunk on alcohol?’; and (iii) ‘Have you ever found yourself in situations you later regretted because of alcohol?’.

#### Progression, cigarettes

Progression of use of cigarettes was categorized as light use ( < six cigarettes on a typical day) and heavier use (≥ six cigarettes on a typical day).

#### Progression, marijuana

Progression of use of marijuana was categorized as light life-time use ( < six times) and heavier life-time use (≥ six times).

For each substance, individuals who had never tried the substance under analysis were coded as ‘missing’ for the progression of use. This was because their position on the progression of use variable was unknown.

### Analyses

As all variables were binary, when assessing the relationship of the variables with age biserial correlations were calculated using the statistical analysis package PRELIS [34].

### Genetic analyses

**Figure 1:**
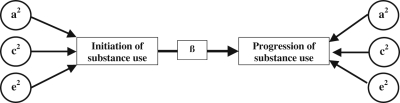
Causal–common–contingent (CCC) model. The model allows the estimation of a^2^ (additive genetic variance), c^2^ (common environmental variance) and e^2^ (unique environmental variance) for initiation and progression of substance use, while controlling for the influence of initiation on progression. This model also allows the modelling of the relationship (beta coefficient value) between the initiation and progression

A CCC model was used for analyses, which allows substance use to be conceptualized as a two-stage process incorporating initiation of substance use and progression to heavier use [23–25]. This model allows the estimation of the magnitude of the relationship between initiation and progression (by means of a beta pathway between these two stages [see Fig. 1]). If the beta coefficient is estimated to be zero, this suggests that the initiation and progression stages are entirely unrelated processes, i.e. genetic and environmental risk factors for initiation are completely independent from those for progression. If the beta coefficient is estimated to be 1, this indicates that initiation and progression are entirely overlapping dimensions with identical genetic and environmental risk factors. The 95% confidence intervals around the beta coefficient provide further information on the degree of overlap between the two stages. Lower limits closer to 0 (or below) support independent liabilities and upper limits approaching 1 provide support for identical liabilities. This model therefore provides a means of testing directly the strength of association between the initiation and progression stages for a substance. It also allows the estimation of: (1) additive genetic effects (a^2^), (2) common environmental effects (c^2^) and (3) unique environmental effects (e^2^) on both initiation and progression of substance use. However, the genetic and environmental influences on progression are estimated after those on initiation have been taken into account. That is, the genetic and environmental influences on progression do not include those on initiation that also effect progression. The amount of variance in progression explained by those influences on initiation can be calculated by squaring the beta coefficient. The proportion of this variance that is explained by genetic factors is equivalent to the proportion of variance in initiation explained by genetic factors, and similarly for the environmental factors.

Another important feature of the model is that it is uniquely suited to analysis of data from adolescent samples, some of whom may have yet to engage in substance use but who will go on to develop problem use. The position of those yet to initiate on the liability distribution of progression is unknown. These individuals are treated as a special case of missing data on the measure of progression [23]. Given the likelihood of an association between age and level of substance use, an age correction was used which adjusts the threshold for each twin according to their age on the distribution of liability to substance use. The threshold is modelled as a simple linear function: *t*_*i*_ = *t* + age_*i*_*t*_a_, where *t* is the population baseline threshold (for individuals of age zero), *t*_a_ models the regression of the threshold on age and age_*i*_ is the age in years of the individual *i* at assessment [23]. The thresholds were also allowed to vary according to gender. However, there was no significant difference between models with and without a gender covariate. This estimate was therefore dropped in favour of the more parsimonious model presented. All structural equation modelling was performed with the software package Mx, using full information maximum likelihood estimation with raw ordinal data [35].

## RESULTS

Prevalence of substance involvement in our sample was broadly in line with rates reported previously in another UK-based sample, ESPAD [9], of 15–16-year-olds. ESPAD reported rates of life-time use of alcohol at 91%, with 68% having been drunk in the last year. In our sample for the same age group the rates were 91% and 57%, respectively. For life-time cigarette use and marijuana use ESPAD reported prevalence rates of 58% and 35%, while we found rates of 50% and 24% for the same age group.

### Alcohol use

A total of 1747 individuals (86%) reported having a drink at some point in their life. Of these, the majority reported only light use of alcohol rather than heavy use (Table 1). Approximately only one-third of those who reported drinking also reported binge drinking, getting drunk or getting into situations they regretted in the last year (Table 1).

**Table 1:** Alcohol use and heavy use within the sample, split by legal age of use.

	Under age 18	18 or over	Total sample
Never had a drink	263 (16%)	22 (5%)	285 (14%)
Light use (≤ 10 drinks in a normal week during the last year)	1241 (92%)	273 (69%)	1514 (87%)
Heavy use (> 10 drinks in a normal week during the last year)	108 (8%)	125 (31%)	233 (13%)
Engaged in binge drinking (≥ 5 drinks in a row more than twice in the last year)	241 (18%)	215 (54%)	466 (27%)
Getting drunk (got drunk more than twice in the last year)	337 (25%)	242 (61%)	595 (34%)
Regretting situations (been in situations later regretted due to alcohol once or more)	348 (26%)	231 (58%)	579 (33%)

With the exception of the percentage of individuals who have never had a drink, all percentages are with regard to the number of individuals who have reported having a drink at some point in their life.

A greater percentage of individuals who were aged over 18 years old (the age at which it is legal to purchase alcohol in the United Kingdom) had tried alcohol compared to those under age 18 (Table 1). Age was correlated significantly with all alcohol-related outcomes, including heavy use (*r* = 0.50), binge drinking (*r* = 0.51), getting drunk (*r* = 0.52) and getting into situations later regretted due to alcohol (*r* = 0.38). None the less, the majority of individuals (83%) under 18 years old also reported use of alcohol.

### Cigarette use

A total of 902 individuals (58%) reported having tried cigarettes. Of these, the majority reported only light use (Table 2). A larger percentage of individuals reported smoking when over the legal age (16 years) than those under the legal age (Table 2) and there was a significant correlation between age and number of cigarettes smoked (*r* = 0.29).

**Table 2:** Cigarette use and heavy use within the sample, split by legal age of use.

	Under 16	16 years or over	Total scores
Never smoked a cigarette	751 (64%)	373 (43%)	1124 (55%)
Light use of cigarettes ( < 6 cigarettes in a normal day during the last month)	341 (82%)	343 (70%)	684 (76%)
Heavy use of cigarettes (≥ 6 cigarettes in a normal day during the last month)	73 (18%)	145 (30%)	218 (24%)

With the exception of the percentage of individuals who have never tried a cigarette, all percentages are with regard to the number of individuals who have reported having a cigarette at some point in their life.

### Marijuana use

Of 438 individuals (22%) who reported having tried marijuana, 278 (62%) reported light use ( < six times during their life) and 160 (38%) reported heavier use (≥ six times during their life). There was a significant correlation between age and life-time use (*r* = 0.28)

### Genetic analyses

Results of the CCC analysis are presented in Table 3.

**Table 3:** Standardized genetic, environmental and beta estimates and confidence intervals for the causal–common–contingent (CCC) multivariate model.

	Initiation of substance use				Progression of substance use			
			
Substance	a^2^ (95% CI)	c^2^ (95% CI)	e^2^ (95% CI)		beta (β) (95% CI)	a^2^ (95% CI)	c^2^ (95% CI)	e^2^ (95% CI)
Alcohol	0.26 (0.04–0.50)	0.65 (0.42–0.83)	0.09 (0.04–0.17)	Quantity	0.48 (0.02–0.72)	0.64 (0.12–0.77)	0.00 (0.00–0.00)	0.36 (0.14–0.46)
				Binge drinking	0.52 (0.19–0.76)	0.38 (0.14–0.60)	0.18 (0.00–0.41)	0.44 (0.30–0.49)
				Getting drunk	0.65 (0.35–0.81)	0.27 (0.15–0.49)	0.36 (0.00–0.50)	0.37 (0.15–0.38)
				Regretting situations	0.52 (0.20–0.74)	0.41 (0.10–0.65)	0.16 (0.00–0.45)	0.43 (0.20–0.47)
Cigarettes	0.41 (0.30–0.74)	0.42 (0.12–0.61)	0.18 (0.10–0.36)	Quantity	87 (0.75–1.00)	1.00 (0.41–1.00)	0.00 (0.00–0.10)	0.00 (0.00–0.05)
Marijuana	0.35 (0.05–0.63)	0.47 (0.24–0.71)	0.18 (0.10–0.36)	Quantity	0.88 (0.38–0.99)	0.64 (0.00–0.65)	0.00 (0.00–0.00)	0.36 (0.00–0.48)

Standardized parameter estimates for the sources of variation in liability to initiation and progression of alcohol, cigarette and marijuana use and a measure of the relationship between initiation and progression (β) are provided. a^2^, additive genetic variance; c^2^, common environmental variance; e^2^, unique environmental variance; beta causal pathway between initiation and progression. Full models with 95% confidence intervals (CI).

#### Alcohol use

For initiation of alcohol use, common environment was the most important factor (c^2^ = 65%) while genetic influences contributed 26%, with little evidence for unique environmental influences (e^2^ = 9%). For quantity of use, there was no evidence of specific common environmental influences, with genetic factors explaining most of the variance (a^2^ = 64%). For the other indices of alcohol progression (binge drinking, getting drunk and getting into situations regretted due to alcohol), the influence of specific genetic factors onprogression tended to be stronger than the influence of genetic factors on initiation of alcohol use, while estimates of common environmental influences tended to be lower. The beta coefficients between initiation and progression (which is a measure of the degree of relationship between the underlying liabilities) for all alcohol outcomes were moderate in size (α = 0.48–0.65). Between 23% and 42% of the variance of progression was due to factors influencing initiation. These findings suggest partial but not complete overlap between the liabilities for alcohol initiation versus progression to more serious use. Of the variance of progression explained by factors influencing initiation, 65% was due to shared environmental factors, 26% by genetic factors and 9% by non-shared environmental factors.

#### Cigarette use

For initiation of cigarette use, common environmental and genetic influences were of equal importance (a^2^ = 41%, c^2^ = 42%). Genetic factors explained almost the entire variance specific to progression (a^2^ = 100%), with unique environmental factors explaining very little variation (e^2^ < 0.1%). The beta coefficient between initiation and progression was high (α = 0.87) and the confidence interval included 1, implying considerable overlap in the initiation and progression liabilities for cigarette use. Of the variance of progression, 76% was explained by factors influencing initiation and of the variance of progression explained by factors influencing initiation, 42% was due to shared environmental factors, 41% by genetic factors and 17% by non-shared environmental factors.

#### Marijuana use

For initiation of marijuana use, common environment explained the largest proportion of variance (c^2^ = 47%), although genetic factors also played a significant role (a^2^ = 35%). For quantity of marijuana use, both genetic (64%) and unique environmental (36%) factors played a role. The confidence intervals around both these estimates included 0; however, they could not both be dropped from the model simultaneously without a significant deterioration in fit. The beta coefficient was high (α = 0.88), with the upper limit of the confidence interval approaching 1 (upper CI = 0.99), again suggesting considerable overlap of liabilities for initiation and progression. Of the 77% of the variance of progression that was explained by factors influencing initiation, 47% was due to shared environmental factors, 35% by genetic factors and 18% by non-shared environmental factors.

## DISCUSSION

A new approach was used to investigating the relationship between the initiation and progression of substance use and applied here for the first time in an adolescent sample. The approach we used facilitated the assessment of genetic contributions to initiation and, importantly, to progression while controlling for age and the influence of substance use initiation on progression.

Prevalence rates in the present study demonstrated high levels of adolescent use of cigarettes, alcohol and marijuana, and are comparable with other UK-based studies [9]. This suggests that our study sample is generally representative of adolescents and young people living in the United Kingdom.

The method of analysis employed in the present study facilitated examination of the relationship and degree of overlap between the liabilities to initiation and progression of substance use. Differences were observed between alcohol use and cigarette and marijuana use. The relationship between alcohol initiation and heavy/problem use was found to be moderate in magnitude (range of beta coefficients: 0.48–0.65). Overall, the results concur with findings from previous twin studies in adults [17], suggesting separate but related liabilities for initiation of use and frequency of alcohol use.

For cigarette use, a beta value of 0.87 and an upper confidence interval including 1 provided evidence that the underlying liabilities for initiation and quantity of use may lie on the same continuum. Heavier use would then represent a higher loading on the same liability distribution as initiation. This suggests that there may be substantial overlap in the risk factors involved in both initiation and progression. It does not mean that individuals who initiate smoking will necessarily progress to heavier use. However, nicotine is a highly addictive substance [36] and it is possible that this is reflected in the strong relationship we find between initiation and progression. For marijuana use, we also found evidence for mainly overlapping liabilities [beta value of 0.88 and an upper confidence interval approaching unity (0.99)]. This suggests that the risk factors implicated in initiation may also be important for progression to heavier use. Longitudinal, epidemiological studies of cigarette and marijuana use in adolescents in a US-based sample also suggest considerable overlap in risk factors (e.g. peer substance use and other substance use) for the initiation and progression stages, with only a few stage-specific risk factors found [37,38].

We examined the genetic and environmental influences on initiation of substance use and progression to heavier substance use (while controlling for the influences of initiation on progression). The pattern of results indicated that environmental factors (particularly common environment) were most important in the initiation of substance use. However, sibling interaction processes could, in part, explain this finding (i.e. with one twin's substance use influencing the substance use of the other twin and vice-versa [13]). In the progression to heavier/problem use, genetic and unique environmental influences appeared to be of greater importance. The exception in this study was cigarette use, for which genetic factors appeared equally important for initiation of use as common environmental influences.

It seems plausible that environmental factors, such as accessibility (availability and cost), societal, familial and peer norms and values, are more important in substance initiation. For example, in the United Kingdom the great majority of people first drink alcohol during their teens and consumption peaks in the late teens and early 20s [9,39]. Those who do not drink will usually not do so for cultural reasons, such as religious faith. For those without such cultural reasons to abstain, some alcohol consumption is ‘normal’. The increased importance of genetic factors with heavier use may reflect biological processes involved in the adaptation and habituation of the brain and body. Animal studies have indicated that the process of addiction depends on the dysregulation of neurochemical mechanisms in specific brain reward and stress circuits [40,41].

Previous studies of adult cigarette and marijuana use, adopting the CCC model approach, found a similar pattern of results to the present study [24,25]. Maes and colleagues [24] reported that the majority of the variance in quantity of cigarette use was explained by factors influencing initiation of cigarette use. However, unlike the present study, they reported that the beta coefficient could not be set to 1 without a significant drop in model fit, and concluded that two separate but related liabilities for initiation and progression characterized their data most effectively.

Agrawal and colleagues [25], when examining adult marijuana use, found a comparably high beta value (β = 0.86) and upper confidence interval (0.98) to the present study. The similarity in the pattern of results is interesting, given that our sample included adolescents who had not yet reached the age of risk of initiation or progression and that we took a relatively broad cut-off point in the quantity of use. This may indicate that similar processes underlie the relationship between initiation and progression of substance use for adolescents and adults.

Most epidemiological risk factor studies have focused upon only one stage of substance involvement (usually initiation [38]). Our findings in adolescents, together with those of several previous adult twin studies, indicate that initiation and progression of substance use are partially independent liabilities (particularly in the case of alcohol). Epidemiological studies providing greater insight into the extent to which risk factors influence initiation and progression stages of substance involvement separately, or in common, are important for prevention and intervention approaches.

### Implications for policy

It is necessary to be cautious in the interpretation of the implications of these results for policy, as this study represents analysis from one, albeit relatively large, population-based sample at one time-point. It is also important to view the findings in the context of the known life-course for each of the three substances in the United Kingdom. For alcohol use, the moderate degree of relatedness between initiation and quantity/problem use suggests that research may benefit from looking for risk factors specific to the development of quantity/problem use of alcohol rather than that of initiation. It may be more appropriate to develop interventions based on these risk factors rather than attempting to reduce initiation of alcohol use. This is particularly the case given the high proportion of twins who have tried alcohol (86%), and the much smaller proportion of that group who reported heavy use of alcohol (26%). This suggests it may be difficult and unrealistic to reduce initiation of alcohol use during adolescence, given that it is the period during which the vast majority of the population first tries alcohol.

Given that approximately half of all individuals who try cigarettes become smokers and half of these die young from cigarette-related health problems [42], primary prevention has long been argued to be the best form of intervention. This twin study supports this argument by finding that the risk of progression upon initiation is great, suggesting that the underlying aetiological risk factors involved in initiation of cigarette use appear to be predominantly the same as those for heavier, more serious use.

The findings for marijuana use were similar to those for smoking, indicating that the same underlying aetiological factors are responsible for initiation and quantity of use. However, these results and their implications for policy must be interpreted tentatively, given the comparatively small number of individuals who reported marijuana use in our sample. Experimentation with drug use typically peaks at the end of adolescence [1] and the number of individuals who continue to use marijuana during adulthood declines with age. Therefore, our results should be interpreted in the context of other research into marijuana use and take into account the differences in life-course use of marijuana and cigarettes.

### Limitations

Several limitations are noteworthy. First, a large part of the sample is below the age of risk for initiation and progression to heavier use for each substance. This limitation is perhaps of most consequence to the analysis of marijuana use, as relatively few individuals (8%) in this sample had progressed to heavier use. However, adolescence is a key period for the initiation of substance use and one aim of the study was to examine the influences on initiation and progression specifically within this age group. We employed statistical methods that were appropriate for this specific sample. Given that progression to clinical dependence on any of the substances studied could not be examined, this leaves open the question of the relationship between progression from heavier use to clinical dependence.

The model tested in the present study included measurement error as part of the beta coefficient estimate. This may have inflated the magnitude of the beta coefficient and thus contributed to the strong relationship between initiation and progression. This may explain partially why almost no unique environmental influence was estimated for quantity of cigarettes smoked and the estimate for unique environmental influence on quantity of marijuana use was non-significant, as the majority of unique environmental influences for progression, including measurement error, could be shared with the initiation of these substances use.

The CCC model assumes that the genetic and environmental influences on initiation influence progression via the beta pathway (Fig. 1). It is also plausible that, for example, it is only genetic influences on initiation that influence progression. Simulation work by Heath and colleagues [43] suggests that initiation and progression estimates and the size of the relationship between initiation and progression are estimated correctly using the CCC approach, but that the genetic and environmental correlations between initiation and progression can be biased. However, their alternative requires at least three categories in the initiation stage, of which at least two lead to information being available on progression, and this may not always be appropriate. Information that would have allowed the coding of initiation in this manner was not available, but would provide an interesting and important extension to the present study.

## CONCLUSION

It appears that there is an underlying aetiology of alcohol problem use that overlaps partially with initiation of alcohol use. Given the high prevalence of initiation of alcohol use, it may represent a more cognisant use of resources to target interventions at the development of more serious alcohol use rather than trying to prevent alcohol use completely within this age group. However, the results from analyses of both marijuana use and cigarette suggest that it may be more important to intervene to stop initiation of use, as the risk factors influencing initiation will also influence progression to more serious use.
